# Miscellaneous

**Published:** 1888-11

**Authors:** 


### MISCELLANEOUS.

Diet for Liver Complaint.—At once discontinue the use of rich, oily, greasy and sugary foods, and of milk and food made with milk in every form. Also use heavy meats as little as possible ; take butchers' meat only two or three times a week, and then very little. Such heavy fowls as ducks and geese, and rich, oily fish, such as salmon, mackerel and eels, should also be discarded. You must not have alcohol in any form if you do not want to weaken your liver. It is the weakening of the liver by alcohol and hot and sloppy liquids, that prevents the liver from doing its work. If you ask what you are to eat, there is no difficulty. You can have most green garden stuff, not much turnip or carrot ; you have cereals, rice, oatmeal, etc., and home-made bread (not very much bread, however), fish, fruit (if eaten as food, not after a heavy meal). Drop tea and coffee, and all hot liquids ; take a glass of spring water when getting up early in the morning, and another when going to bed. Chew your food slowly ; masticate well. Do not drink at meals, or at the morning meal drink one small cup of freshly made tea only, but you had better be without it. You will find that you enjoy your food just as well without liquid, and that the biliousness will gradually disappear if you persevere. Take no medicine of any kind, except in the shape of diet.

Names of Chemicals.—While scientific names are useful and necessary, their employment in published recipes sometimes causes a good deal of mystification to people otherwise well informed.—"Where can I get sodic chloride ?" said a lady to the writer once. She was seven miles from a drug store, and her hope was that I might have this "rare" chemical among my stock used for experimental purposes. "Go to the salt barrel," was the answer : you will find it there." Common salt also goes by the names chloride of sodium and muriate of soda. When Dr. Dosem sends you to the pharmacy with the prescription Potass. nit., he believes that saltpeter is good for rheumatism. When he writes Sod. bicarb, you probably have sour stomach which he thinks baking soda may relieve. Copperas, green vitriol, ferrous sulphate and sulphate of iron are all the same. White vitriol is sulphate of zinc ; blue vitrol, sulphate of copper ; oil of vitriol, sulphuric acid. Sulphate of magnesia and Epsom salts are the same. Gypsum is sulphate of lime, otherwise calcic sulphate.

Russian Sheet Iron.—Probably the only secret process which has been kept inviolate, and for ages openly defied the world of science, is the iron trade of Russia. The secret of making Russia sheet iron is owned by the Government, and is such an immense monopoly that it is currently supposed to defray the entire expenses of the Government.

The works constitute an entire city, isolated and fortified against the rest of the world. When a workman enters the service he bids a last farewell to his family and friends, and is practically lost to the world. He is never heard from afterward, and whether he lives or dies, all trace of him is forever lost.

There have been several desperate attempts made to steal or betray the secret, but in every instance it has resulted in the death of the would-be traitor.

In one case a letter attached to a kite, which was allowed to escape, was picked up by some peasants, and, despite their protestations that they were unable to read, they were at once put to death by the guards to whom they delivered the letter, and it was afterward decreed that the guards themselves should pass the remainder of their days within the works.

The wonderful properties of this iron are so well known that it is unnecessary to enlarge upon them ; imitations have been made closely resembling the original article, but the durable polish, toughness and anti-rusting properties are lacking, and to-day the secret remains as hidden as the philosopher's stone.

Lord Rosse's Telescope.—The late Lord Rosse erected a very large telescope upon his estate at Birr Castle, near Parsontown, in Ireland. The tube of the instrument is fifty-five feet long, and weighs six and one-half tons. With Lord Rosse's telescope we can see the surface of the moon so much enlarged that a space 220 feet long could be readily perceived by a good observer, and it is certain that if a troop of buffaloes or animals similar to them crossed the field of vision they would undoubtedly be visible. Masses of troops marching backwards and forwards would also be plainly seen, and we may assert with something like absolute certainty, that there are neither towns nor villages in the moon, nor any buildings as large as our own St. Paul's, or as large as our chief railway stations.—*The Naturalist's Note Book.*

Mr. Kennan's Change of Views.—The following is an extract from a letter written home by Mr. Kennan while he was in Russia investigating the exile system for *The Century* magazine :

"When I write up this country for *The Century*, I shall have to take back some of the things that I have said. The exile system is worse than I believed it to be, and worse than I have described it. It isn't pleasant, of course, to have to admit one has written upon a subject without fully understanding it ; but even that is better than trying, for the sake of consistency, to maintain a position after one sees that it is utterly untenable."

### MY LOVE.

My love is a milliner's daughter,
And lives right over the way,
A shiny new thimble I've bought her ;
I'm sure she'll never say nay.

All the day she's fashioning laces,
And ribbons that yet are to be
The wreathings of bright, happy faces,
But Fanny's the sweetest to me.

O ! little she wots, when demurely,
She's plying her needle so true,
That willie or nillie, as surely,
She's stitching my heart through and through.

La Croix.

